# Quality of the documentation of the Nursing process in clinical decision support systems*

**DOI:** 10.1590/1518-8345.4510.3426

**Published:** 2021-05-21

**Authors:** Neurilene Batista de Oliveira, Helosa Helena Ciqueto Peres

**Affiliations:** 1 Universidade de So Paulo, Hospital Universitrio, So Paulo, SP, Brazil.; 2 Universidade de So Paulo, Escola de Enfermagem, So Paulo, SP, Brazil.

**Keywords:** Decision Support Systems, Clinical, Nursing Process, Standardized Nursing Terminology, Electronic Health Records, Nursing Informatics, Technology Assessment, Biomedical, Sistemas de Apoio a Decises Clnicas, Processo de Enfermagem, Terminologia Padronizada em Enfermagem, Registro Eletrnico em Sade, Informtica em Enfermagem, Avaliao de Tecnologias de Sade, Sistemas de Apoyo de Decisiones, Clnicos, Proceso de Enfermera, Terminologa Normalizada de Enfermera, Registros Electrnicos de Salud, Informtica Aplicada a la Enfermera, Evaluacin de la Tecnologa Biomdica

## Abstract

**Objective:**

to compare the quality of the Nursing process documentation in two versions of a clinical decision support system.

**Method:**

a quantitative and quasi-experimental study of the before-and-after type. The instrument used to measure the quality of the records was the Brazilian version of the *Quality of Diagnoses, Interventions and Outcomes*, which has four domains and a maximum score of 58 points. A total of 81 records were evaluated in version I (pre-intervention), as well as 58 records in version II (post-intervention), and the scores obtained in the two applications were compared. The interventions consisted of planning, pilot implementation of version II of the system, training and monitoring of users. The data were analyzed in the R software, using descriptive and inferential statistics.

**Results:**

the mean obtained at the pre-intervention moment was 38.24 and, after the intervention, 46.35 points. There was evidence of statistical difference between the means of the pre- and post-intervention groups, since the p-value was below 0.001 in the four domains evaluated.

**Conclusion:**

the quality of the documentation of the Nursing process in version II of the system was superior to version I. The efficacy of the system and the effectiveness of the interventions were verified. This study can contribute to the quality of documentation, care management, visibility of nursing actions and patient safety.

## Abstract (pt)

**Objetivo:**

comparar a qualidade da documentao do processo de enfermagem em duas verses de um sistema de apoio deciso clnica.

**Mtodo:**

estudo quantitativo, quase-experimental do tipo antes e depois. O instrumento utilizado para mensurar a qualidade dos registros foi o *Quality of Diagnoses, Interventions and Outcomes* Verso Brasileira, que possui quatro domnios e escore mximo de 58 pontos. Foram avaliados 81 registros na verso I (pr-interveno), 58 registros da verso II (ps-interveno) e comparados os escores obtidos nas duas aplicaes. As intervenes consistiram em planejamento, implantao piloto da verso II do sistema, treinamento e acompanhamento dos usurios. Os dados foram analisados no *software* R, utilizando-se estatstica descritiva e inferencial.

**Resultados:**

a mdia obtida na pr-interveno foi de 38,24 e ps-interveno, 46,35 pontos. Houve evidncias de diferena estatstica entre as mdias dos grupos pr e ps-interveno, visto que o valor-p foi menor que 0,001 nos quatro domnios avaliados.

**Concluso:**

a qualidade da documentao do processo de enfermagem na verso II do sistema foi superior verso I. A eficcia do sistema e a efetividade das intervenes foram comprovadas. Este estudo pode contribuir para a qualidade da documentao, gerenciamento do cuidado, visibilidade das aes de enfermagem e segurana do paciente.

## Abstract (es)

**Objetivo:**

comparar la calidad de la documentacin del proceso de Enfermera en dos versiones de un sistema de apoyo a la decisin clnica.

**Mtodo:**

estudio cuantitativo y cuasi-experimental del tipo antes y despus. El instrumento que se us para medir la calidad de los registros fue la versin brasilea del *Quality of Diagnoses, Interventions and Outcomes*, que contiene cuatro dominios y puntaje mximo de 58 puntos. Se evaluaron 81 registros en la versin I (pre-intervencin), 58 registros de la versin II (post-intervencin) y se compararon las puntuaciones logradas en las dos aplicaciones. Las intervenciones consistieron en planificar, implementar de forma piloto la versin II del sistema, entrenar y realizar un seguimiento a los usuarios. Los datos se analizaron en el *software* R, utilizando estadstica descriptiva e inferencial.

**Resultados:**

el promedio obtenido en el momento pre-intervencin fue de 38,24 y en la post-intervencin fue de 46,35 puntos. Se registraron evidencias de diferencia estadstica entre los promedios de los grupos pre- y post-intervencin, visto que el valor-p fue menor que 0,001 en los cuatro dominios evaluados.

**Conclusin:**

la calidad de la documentacin del proceso de Enfermera en la versin II del sistema fue superior a la versin I. Se comprob la eficacia del sistema y la efectividad de las intervenciones. Este estudio pudo contribuir para la calidad de la documentacin, gerenciamiento de la atencin, visibilidad de las acciones de Enfermera y seguridad del paciente.

## Introduction

The Nursing Process (NP) systematizes the clinical evaluation, evidences clinical reasoning, decision-making and clinical judgment about the patients responses to life problems and processes. It is a methodological tool for the planning of assistance and documentation of the care plan. In addition, it integrates and organizes information, facilitates communication between the members of the interdisciplinary team, and contributes to quality of care and patient safety^(^ 1 ^)^.

NP appeared in the United States in the 1950s, initially as a guide for nursing students. It was later adopted in the nursing services to give greater autonomy to the profession, to favor holistic care, and to provide continuity of care^(^ 2 ^)^.

In Brazil, from the work of Horta (1979), the NP was gradually inserted in the undergraduate curricula in Nursing and in the care practice. From Resolution 358/2009of the Federal Nursing Council (*Conselho Federal de Enfermagem*, COFEN), its application^(^ 3 ^-^ 4 ^)^ has become mandatory in all the environments where professional nursing care occurs.

However, documenting the stages of the NP is still a challenge for health institutions. A survey on the prevalence of documentation in hospitals, clinics and outpatient clinics administered by the Health Secretariat of the State of So Paulo showed that, of the 416 sectors studied, only 288 (69.3%) recorded four stages of the NP^(^ 5 ^)^.

The NP documentation requires nurses to have knowledge about standardized concepts, rooted in scientific bases of the Nursing Classifications, also known as Standardized Language Systems (SLS)^(^ 6 ^)^. These systems provide the framework for organizing important concepts about diagnoses, interventions and outcomes. It even serves as a guide for the NP to be documented in unambiguous language, assists in clinical reasoning, manages care, inter-professional communication and decision-making^(^ 7 ^)^.

SLSs are a prerequisite for the construction of Electronic Health Records (EHRs), aiming to retrieve information for research studies, statistical analysis, benchmarking, big data, data interoperability between the different health systems and, above all, to ensure continuity and quality of care^(^ 6 ^)^.

However, finding essential patient data from the perspective of Nursing in the EHRs or in the printed medical records is a major challenge. Research studies show that the quality of Nursing documentation varies from moderate to poor. This includes lack of information, limitations in diagnostic accuracy, inaccurate and redundant documentation^(^ 8 ^-^ 10 ^)^. Both the EHR and the paper record have problems related to content, process and structure criteria^(^ 5 ^,^ 11 ^)^.

Nursing can benefit from the development and implementation of EHRs with SLS and Clinical Decision Support Systems (CDSSs). Decision support is a resource that provides access to clinical guidelines and protocols based on scientific evidence. In addition, it can support nurses in NP documentation and in the formulation of accurate diagnoses and effective interventions, which can contribute to highly significant and clinically relevant outcomes for the patient^(^ 6 ^,^ 12 ^)^.

In this context, the University Hospital of the University of So Paulo (*Hospital Universitrio da Universidade de So Paulo*, HU-USP) in partnership with the School of Nursing of the University of So Paulo (*Escola de Enfermagem da Universidade de So Paulo*, EEUSP), developed a CDSS that had as its premise to support the documentation of the NP, the improvement of clinical reasoning in nurses and students, building evidence bases for the profession and promoting the development of research studies^(^ 13 ^)^.

It is noteworthy that, in the development of this technological tool, there was the active participation of nurses from the HU-USP Nursing Department, EEUSP professors and undergraduate and graduate students. This system has been favoring the improvement of the quality of NP documentation and of assistance, of a humanized, individualized care centered on the client and the family. It was called Electronic Documentation System of the Nursing Process of the University of So Paulo (PROCEnf-USP)^(^ 13 ^)^.

The PROCEnf-USP was organized according to a knowledge base anchored in the definitions of diagnoses and their components and follows the hierarchy of domains and classes, proposed by the unification of the structures of NANDA International (NANDA-I), NOC (Nursing Outcomes Classification) and NIC (Nursing Interventions Classification). This unification of the structures is known as NANDA-NOC-NIC Linkages or NNN Linkages^(^ 13 ^-^ 17 ^)^.

Version I of this system was implemented in 2009 at the Medical and Surgical Clinics, covering three (03) stages of the NP: Data Collection (Nursing History and Physical Examination), Nursing Diagnosis and Planning (Outcomes, without the use of Indicators, and Nursing Interventions). The Nursing Prescription was performed in the system; however, the scheduling and checking were performed in a handwritten manner. Nursing Evolution was conducted by hand, with the Nursing Diagnostics being attributed the following qualifiers: worsened, improved, unchanged and resolved.

PROCEnf-USP Version II was implemented in October 2019, as a pilot project at the HU-USP Surgical Clinic. In this version, the 5 (five) stages of the NP were incorporated, namely: Data Collection (Nursing History and Physical Examination), Nursing Diagnosis, Planning (Outcomes, using the NOC Indicators, Nursing Interventions and computerized scheduling of the Nursing Prescription), Implementation (prescription check and computerized Nursing Annotation), and Nursing Evolution with NOC Outcome Indicators, used to measure the results achieved.

A free text space was also available for version II of the system on the Evaluation Summary screen, in which the nurse can record important information about the patient, discharge guidelines and conditions for intra- and extra-hospital transfer.

It is worth considering that the two versions of the system have mechanisms that prevent the user from progressing in the evaluation if at least one Nursing Outcome, Indicator, Intervention and Prescription activity is not selected for each Diagnosis. These requirements were defined in the system design phase and were adopted both in version I and in version II.

In view of the incorporation of new functionalities, evaluating the usability of this system to document NP is an important challenge and objective, due to the moral and ethical responsibility with users, patients and health professionals. Thus, the minimization of errors, the increase in the quality of care and the safety of patients are guaranteed.

Usability is defined as the extent to which a system, product or service can be used by specific users to attain objectives with effectiveness, efficiency and satisfaction in a specific use context. Effectiveness refers to the precision and integrity with which users attain specific objectives^(^ 18 ^)^.

This study is justified by the need for reliable academic research studies to demonstrate the usability of CDSS in the NP documentation. This is due to the fact that the implementation and deployment studies suggest that functionality and usability affect satisfaction, efficiency and effectiveness in clinical use.

The hypothesis of this study is that the nursing documentation in PROCEnf-USP version II is more effective and has better quality when compared with version I of the system.

In this way, the present study was designed with the objective of comparing the quality of the NP documentation in two versions of a clinical decision support system.

## Method

A quantitative and quasi-experimental study of the before-and-after type. In the quasi-experimental design, also known as field experiment, the researcher limits the influence and control over the selection of the study participants. In this type of study, the researcher cannot randomly assign participants and/or ensure that the selected sample is as homogeneous as desirable^(^ 19 ^)^.

In these surveys, the ability to fully control all the study variables and the implications of the intervention is limited. However, quasi-experimental studies provide fruitful information for advancing research. In addition, in numerous research studies, including those conducted in information systems research, randomization may not be feasible^(^ 19 ^)^.

The effectiveness of the system was determined by the precision and integrity with which the nursing team attained the objective of carrying out the NP documentation in versions I and II of the system. The interventions consisted of planning and implementing version II of the system, as well as in training and monitoring the team.

The study was developed in a public teaching hospital, with secondary care complexity, located in the West of the city of So Paulo. The unit selected to carry out the study and pilot implementation of version II of the system was the Surgical Clinic, an inpatient unit for adult patients, which has used version I of the system since 2009 and has the NP documentation strongly consolidated, being able to have the implementation results replicated in other units of the hospital.

The Surgical Clinic has 27 beds, with an annual occupancy rate of 89%. The staff is composed of 14 nurses, including the head of the section and the researcher, 28 nursing technicians/assistants, two of whom were on sick leave, one was in the process of rehabilitation and one was carrying out activities related to materials management.

The instrument used for data collection was the Brazilian version of *Quality of Diagnoses, Interventions and Outcomes* (Q-DIO), which consists of 29 items distributed in four subscales, each of which is scored on a three-point scale (0=Not documented, 1=Partially documented, and 2=Complete documentation). The minimum score is zero and the maximum is 58 points^(^ 20 ^)^.

The Nursing Diagnosis subscale as a process comprises 11 items, with a maximum score of 22 points, and addresses the accuracy of the nursing assessment related to Data Collection (patients history and physical examination)^(^ 20 ^)^.

The Nursing Diagnosis subscale as a product comprises eight items, with a maximum score of 16 points and addresses the accuracy of the nursing diagnoses when using standardized language or the precision of nursing problems, signs and symptoms when using standardized language^(^ 20 ^)^.

The Nursing Interventions subscale comprises three items, with a maximum score of six points, and addresses the effectiveness of nursing interventions on the etiology of the nursing diagnosis/problem^(^ 20 ^)^.

The Nursing Outcomes subscale comprises seven items, with a maximum score of 14 points, and measures the quality of the outcomes of patients sensitive to Nursing^(^ 20 ^)^.

As recommended in the tutorial for using the Brazilian version of Q-DIO, to compose the sample of the first stage a priority diagnosis was selected, relating it to the interventions and outcomes^(^ 20 ^)^.

Thus, it was decided to apply the Q-DIO to the Impaired Tissue Integrity nursing diagnosis, as it is first in the ranking of the ten (10) most frequent nursing diagnoses in the Surgical Clinic. This data was obtained from a report generated by Business Intelligence infoView PROCEnf-USP.

According to the Power Test Analysis, it was estimated that, regarding the minimum sample size, to confidently detect the relevance of the intervention effect, the Q-DIO should be applied to at least 10 nursing records at each stage.

For the selection of the sample of version I of the system, a report was issued regarding the hospitalizations that occurred in the Surgical Clinic in the years 2017 and 2018. The sample consisted of 81 nursing records that had a diagnosis of Impaired Tissue Integrity, whose patients length of stay was at least four days, as recommended by Q-DIO.

Likewise, in order to select the sample for version II of the system, a report was issued regarding the admissions to the Surgical Clinic in the period from November 15^th^, 2019 to January 10^th^, 2020.The sample consisted of 58 nursing records, which had a diagnosis of Impaired Tissue Integrity, whose length of stay was at least four days.

The data were collected by the researcher, in a private environment, ensuring the confidentiality of the information contained in the evaluated medical records. The quality of the records was assessed using the Brazilian version of the Q-DIO instrument, which measures the quality of the documentation and the links between Nursing Diagnoses, Outcomes and Interventions.

Prior to data collection, a pilot test was performed applying the Q-DIO to 10 nursing records. The first stage of the collection took place in the first half of 2019, retrospective to the years 2017 and 2018, referring to the records made in version I of the system. The second stage of the collection took place in January 2020, retrospective to the period from November 15^th^, 2019 to January 10^th^, 2020, referring to the records made in version II of the system.

It should be noted that the records from version I were partially computerized and were collected from the electronic system and from the printed medical records. The records from version II were fully computerized and collection was conducted online in the electronic system.

These records were made by all the nurses and nursing technicians working in the Surgical Clinic. Sociodemographic data were not collected from the patients or from the professionals who performed the records because the objective is not to compare the performance of professionals or establish subdivisions in groups.

The interventions consisted in planning and implementing version II of the system, as well as in training and monitoring of users. To plan the pilot implementation of version II of the system, some concepts were used from the PMBOK Guide - A Guide to the Project Management Body of Knowledge, developed by the Project Management Institute (PMI)^(^ 21 ^)^.

This is a standardization that identifies and conceptualizes processes, areas of knowledge, tools and techniques for project management. According to PMBOK, the phases of a project are as follows: Initiation, Planning, Execution, Monitoring and Control, and Closure^(^ 21 ^)^.

In the Initiation phase, the project and the people involved (stakeholders) were defined, as well as a survey was conducted of the initial risks, which could be evident threats to the project. The main risks identified, which could have a negative impact on the project, were related to human and technological resources.

During this work, an economic crisis was experienced that reflected in the commitment of investments in human resources, reforms and maintenance of the physical structure, as well as in the technological updating of the hospital.

As is public knowledge, the University of So Paulo adopted in 2015 and 2017 the Incentive Program for Voluntary Dismissal (IPVD), as well as defined guidelines to be followed until 2022, represented by Economic Sustainability Parameter - Resolution No. 7344, 2017^(^ 22 ^)^.

These factors implied a reduction in the nursing staff of the hospital, mainly in the number of nursing technicians. In the unit under study, several nursing technicians joined the IPVD, increasing the workload for the team. Therefore, this risk could not be managed at the time of data collection.

Another risk identified refers to the insufficient number of computers to use the software in daily tasks, mainly because it is a teaching hospital, with a large circulation of nursing, medical and resident students.

This risk was managed by the provision of two (02) notebooks installed in two carts at the bedside, exclusive for the nursing team, which remained in the unit during the pilot implantation, for testing and evaluation. These carts were provided by two different companies and remained in the Surgical Clinic during the testing and evaluation period. The notebooks were provided by the institutions Nursing Department.

In the Planning phase, the Analytical Structure of the Project was created, in which the activities that would be conducted to carry out the pilot implementation were defined, such as: preparation of training sessions and tutorials, preparation of the environment, installation of the software in the computers, availability of carts at the bedside with notebooks, training, communication plan and face-to-face follow-up in the post-deployment period.

In the Execution phase, the training sessions for the professionals and the online tutorial videos on prescription checking, nursing evolution and the use of NOC outcome indicators were developed.

The training sessions carried out by the researcher, with support from the hospitals Teaching and Quality Service and IT team, from September 9^th^ to September 20^th^, 2019. There were 9 training sessions lasting 2 hours each, outside working hours, individualized by professional category, and composed of a theoretical and a practical part, in which the professionals used version II of the system. Fictitious patients were created to carry out the practical training, conducted in the academic environment of the system under approval.

In the links below you can view the training script for nurses and nursing technicians:

https://drive.google.com/file/d/15KL8gkTeegwgQNntdrBVkSFoQ1mYLTUH/view?usp=sharing

https://drive.google.com/file/d/1c4VPSzFQQrUHCbCBHMoIE1qSgOMNmijx/view?usp=sharing

In the link below you can access the demonstration video of version II of the PROCEnf-USP system, where you can view the step-by-step documentation of the stages of the NP and the electronic check of the Nursing Prescription:

https://drive.google.com/file/d/1BTzpe4MVzp8ZMowqet3yiT0H5PXtRuSv/view?usp=sharing

The implementation of PROCEnf-USP Version II took place on 10/28/2019. It was decided to deploy it gradually, first in one of the wards and, after one week, having it implemented in the entire clinic.

In the Monitoring and Control phase, system errors or bugs were identified and forwarded for resolution. All the corrective actions were monitored and controlled through tests in the system.

In the Closure phase, the follow-ups and pilot implementation were completed. The system is in operation and meeting the requirements of the NP documentation.

The data collected referring to the application of the Brazilian version of Q-DIO were stored in two Excel spreadsheets, one for each stage. They were organized and analyzed with the aid of the R software^(^ 23 ^)^. The categorical variables of Q-DIO (not documented, partially documented and complete documentation) were described using absolute and relative frequencies. The continuous and discrete quantitative variables were described by measures of central tendency (mean and median) and dispersion (standard deviation).

In order to estimate the significance of the difference between the means of the pre- and post-intervention groups (control and experimental), hypothesis tests were performed: Welch *t* test with two samples, Brunner-Munzel test, and Wilcoxon-Mann-Whitney test, as appropriate for data distribution. A significance level of 5% was adopted, that is, the results were considered significant if the p-value was below 0.05.

The research was conducted in accordance with the Regulatory Guidelines and Norms for Research Involving Human Beings (National Health Council Resolution 466 of 2012). The confidentiality and secrecy of the data was guaranteed, as well as the non-identification of the participants. The project was approved by the Research Ethics Committee of the University Hospital of the University of So Paulo, under CAAE number: 00636818.0.0000.0076. The Free and Informed Consent Term was used, which confirms the participation of the study subjects.

## Results

A total of 81 and 58 records were evaluated before and after the intervention, respectively. The tables 1 to 5 show the percentage of items not documented, partially documented and with complete documentation at the pre- and post-intervention moments, as well as the scores obtained in the four domains and the total Q-DIO score.

**Table 1:** Distribution of the categorical variables in the Nursing Diagnosis as a Process domain of the Brazilian version of the Quality of Diagnoses, Interventions and Outcomes (Q-DIO) instrument, in the pre-intervention and post-intervention groups. So Paulo, SP, 2020

Items of Nursing Diagnosis as a Process	Variables	Pre		Post		p-value
		n	%	n	%	
1 Current situation that led to hospitalization	Not documented	2	2.47	0	0.00	0.046*
	Partially documented	65	80.25	39	67.24	
	Complete documentation	14	17.28	19	32.76	
2 Anxiety, concerns, expectations and desires related to hospitalization	Not documented	57	70.37	23	39.66	0.001^[Table-fn TFN2]^
	Partially documented	21	25.93	32	55.17	
	Complete documentation	3	3.70	3	5.17	
3 Social situation and environment, circumstances in which you live	Not documented	30	37.04	0	0.00	0.001*
	Partially documented	49	60.49	55	94.83	
	Complete documentation	2	2.47	3	5.17	
4 Facing the current situation with the disease	Not documented	65	80.25	9	15.52	0.001^[Table-fn TFN2]^
	Partially documented	15	18.52	42	72.41	
	Complete documentation	1	1.24	7	12.07	
5 Belief and attitudes towards life (related to hospitalization)	Not documented	18	22.22	5	8.62	0.028*
	Partially documented	63	77.78	52	89.66	
	Complete documentation	0	0.00	1	1.72	
6 Information from the patient and family members/significant people about the situation	Not documented	65	80.25	18	31.03	0.001*
	Partially documented	16	19.75	27	46.55	
	Complete documentation	0	0.00	13	22.41	
7 Gender-related personal intimacy issue	Not documented	81	100.00	58	100.00	1.000*
	Partially documented	0	0.00	0	0.00	
	Complete documentation	0	0.00	0	0.00	
8 Hobbies, leisure activity	Not documented	81	100.00	58	100.00	1.000*
	Partially documented	0	0.00	0	0.00	
	Complete documentation	0	0.00	0	0.00	
9 Important people (for contact)	Not documented	21	25.93	0	0.00	0.001*
	Partially documented	59	72.84	25	43.10	
	Complete documentation	1	1.24	33	56.90	
10 Activities of daily living	Not documented	38	46.91	20	34.48	0.049^[Table-fn TFN2]^
	Partially documented	15	18.52	6	10.34	
	Complete documentation	28	34.57	32	55.17	
11 Relevant nursing priorities according to the assessment	Not documented	0	0.00	0	0.00	1.000*
	Partially documented	0	0.00	0	0.00	
	Complete documentation	81	100.00	58	100.00	

* Fisher' Exact Test;

Pearson' Chi-Square Test

**Table 2:** Distribution of the categorical variables in the Nursing Diagnosis as a Product domain of the Brazilian version of the Quality of Diagnoses, Interventions and Outcomes (Q-DIO) instrument, in the pre- and post-intervention groups. So Paulo, SP, 2020

Nursing Diagnosis as a Product	Variables	Pre		Post		p-value
		n	%	n	%	
12 The nursing problem/diagnosis title is recorded	Not documented	0	0.00	0	0.00	1.000*
	Partially documented	0	0.00	0	0.00	
	Complete documentation	81	100.00	58	100.00	
13 The diagnostic title is formulated and numbered according to NANDA^[Table-fn TFN4]^	Not documented	0	0.00	0	0.00	1.000*
	Partially documented	0	0.00	0	0.00	
	Complete documentation	81	100.00	58	100.00	
14 The etiology is registered	Not documented	6	7.41	0	0.00	0.016*
	Partially documented	3	3.70	0	0.00	
	Complete documentation	72	88.89	58	100.00	
15 The etiology is correct and corresponds to the nursing diagnosis	Not documented	6	7.41	0	0.00	0.016*
	Partially documented	3	3.70	0	0.00	
	Complete documentation	72	88.89	58	100.00	
16 The signs and symptoms are recorded	Not documented	1	1.24	0	0.00	1.000*
	Partially documented	0	0.00	0	0.00	
	Complete documentation	80	98.77	58	100.00	
17 The signs and symptoms are correct and related to the nursing diagnosis	Not documented	0	0.00	0	0.00	1.000*
	Partially documented	0	0.00	0	0.00	
	Complete documentation	81	100.00	58	100.00	
18 The nursing goal is related/corresponds to the nursing diagnosis	Not documented	2	2.47	0	0.00	0.510*
	Partially documented	1	1.24	0	0.00	
	Complete documentation	78	96.30	58	100.00	
19 The nursing goal is attainable by means of the interventions	Not documented	2	2.47	0	0.00	0.510*
	Partially documented	1	1.24	0	0.00	
	Complete documentation	78	96.30	58	100.00	

* Fisher's Exact Test;

NANDA International

**Table 3:** Distribution of the categorical variables in the Nursing Interventions domain of the Brazilian version of the Quality of Diagnoses, Interventions and Outcomes (Q-DIO) instrument, in the pre-intervention and post-intervention groups. So Paulo, SP, 2020

Nursing Interventions	Variables	Pre		Post		p-value
		n	%	n	%	
20 Concrete, clearly named according to the NIC^[Table-fn TFN6]^ interventions and planned	Not documented	1	1.24	0	0.00	0.001*
	Partially documented	13	16.05	0	0.00	
	Complete documentation	67	82.72	58	100.00	
21 The nursing interventions have an effect on the etiology of the nursing diagnoses	Not documented	2	2.47	0	0.00	0.428*
	Partially documented	2	2.47	0	0.00	
	Complete documentation	77	95.06	58	100.00	
22 The nursing interventions performed are recorded	Not documented	2	2.47	0	0.00	0.510*
	Partially documented	1	1.24	0	0.00	
	Complete documentation	78	96.30	58	100.00	

* Fisher's Exact Test;

Nursing Interventions Classification

**Table 4:** Distribution of the categorical variables in the Nursing Outcomes domain of Brazilian version of the Quality of Diagnoses, Interventions and Outcomes (Q-DIO) instrument, in the pre- and post-intervention groups. So Paulo, SP, Brazil, 2020

Nursing Outcomes	Variables	Pre		Post		p-value
		n	%	n	%	
23 Critical diagnostic changes are evaluated daily or shift by shift	Not documented	0	0.00	0	0.00	1.000*
	Partially documented	0	0.00	0	0.00	
	Complete documentation	81	100.00	58	100.00	
24 The nursing diagnosis is reformulated	Not documented	0	0.00	0	0.00	1.000*
	Partially documented	0	0.00	0	0.00	
	Complete documentation	81	100.00	58	100.00	
25 The nursing result is recorded	Not documented	0	0.00	0	0.00	0.001*
	Partially documented	81	100.00	0	0.00	
	Complete documentation	0	0.00	58	100.00	
26 The nursing outcome is observable/measured according to the NOC^[Table-fn TFN8]^	Not documented	0	0.00	0	0.00	0.001*
	Partially documented	81	100.00	0	0.00	
	Complete documentation	0	0.00	58	100.00	
27 The nursing outcome indicates improvement	Not documented	58	71.61	0	0.00	0.001*
	Partially documented	18	22.22	16	27.59	
	Complete documentation	5	6.17	42	72.41	
28 There is a relationship between the outcomes and the nursing interventions	Not documented	4	4.94	0	0.00	0.140*
	Partially documented	1	1.24	0	0.00	
	Complete documentation	76	93.83	58	100.00	
29 The outcomes and the nursing diagnoses are internally related	Not documented	4	4.94	0	0.00	0.140*
	Partially documented	1	1.24	0	0.00	
	Complete documentation	76	93.83	58	100.00	

* Fisher's Exact Test;

Nursing Interventions Classification

**Table 5:** Descriptive measures of the scores obtained at the pre- and post-intervention moments, according to the domains of the Brazilian version of the Quality of Diagnoses, Interventions and Outcomes (Q-DIO) instrument. So Paulo, SP, Brazil, 2020

Q-DIO* domains	Moment	n	Mean	Standard Deviation	Median	p-value
Nursing Diagnosis as a Process	Pre-intervention	81	6.95	1.65	7	< 0.001^[Table-fn TFN10]^
	Post-intervention	58	10.62	2.25	11	
Nursing Diagnosis as a Product	Pre-intervention	81	15.48	1.32	16	< 0.001^[Table-fn TFN11]^
	Post-intervention	58	16.00	0.00	16	
Nursing Interventions	Pre-intervention	81	5.68	0.88	6	< 0.001^[Table-fn TFN11]^
	Post-intervention	58	6.00	0.00	6	
Nursing Outcome	Pre-intervention	81	10.12	1.08	10	<0.001^[Table-fn TFN12]^
	Post-intervention	58	13.72	0.45	14	
Total	Pre-intervention	81	38.24	3.00	39	<0.001^[Table-fn TFN12]^
	Post-intervention	58	46.35	2.29	46.5	

* Q-DIO = Quality of Diagnoses, Interventions and Outcomes;

Two sample Welch t-test;

Brunner-Munzel test;

Wilcoxon-Mann-Whitney test

According to Table 1, a reduction in the number of not documented items is observed in the post-intervention group (version II of the system). There is evidence that there was an improvement in the documentation in items 1 to 6, 9 and 10, given that the p-value was below the pre-established significance level (p<0.05).

There was no difference in the documentation of questions 7 and 8, as they remained undocumented in both stages.

According to Table 2, there is evidence of difference in the quality of documentation in items 14 and 15 of the post-intervention group (version II of the system), given that the p-value was below 0.05.

There was no statistical difference in the other questions evaluated because they were already close to the saturation level reaching 100% in the post-intervention group.

According to Table 3, there is evidence of a difference in the quality of documentation between the two versions of the system in item 20, given that the p-value was below 0.05. There was no statistical difference in the other questions evaluated because they were already close to the saturation level, reaching 100% in the post-intervention group.

According to Table 4, there is evidence of difference in the quality of documentation in items 25, 26 and 27 between the pre- and post-intervention groups, given that the p-value was below 0.05. There was no statistical difference in questions 23,24,28 and 29 because they remained 100% documented in versions I and II of the system.

It can be seen that, in item 27, the percentage of complete documentation was 72.41%, indicating that adjustments are still necessary for the documentation to reach higher levels of completeness.

As can be seen in Table 5, the mean Q-DIO score was 38.24 in the pre-intervention group and 46.35 in the post-intervention group. The p-value was below 0.001 in all the domains evaluated. With the pre-established significance level of 5%, there is statistical evidence that the quality of documentation was better in the post-intervention group.

## Discussion

The Brazilian version of Q-DIO has as its main purpose to evaluate the quality of the documentation and the links between Nursing Diagnoses, Interventions and Outcomes^(^ 20 ^)^. According to the scores obtained in the application, the quality of the documentation was better in version II of the system, and there was a decrease in the percentage of undocumented items in all the domains evaluated.

Version II of the system obtained a mean score of 46.35 points, higher than a cross-sectional study carried out in two centers, in which Center one obtained a mean of 35.46 points and the Center two, 31.72 points^(^ 24 ^)^.

In the Nursing Diagnosis as a Process domain, the score obtained did not reach its maximum score because psychosocial aspects such as support network, description of emotions related to the health-disease process, use of coping strategies, and knowledge of the patient and family about the treatment, as well as aspects related to sexuality, hobbies and leisure activities, were partially documented or not documented.

The registration of this information is relevant, as from the identification of these aspects, the nurse is able to predict, detect, prevent and manage real or potential problems, clarify doubts and guide patients and families, in addition to allowing for the identification of patients in situations of social vulnerability that may require specific actions by nurses.

The nursing documentation must contain clinical content relevant to the location and clinical status of the patient, enabling the recording of health status, needs and responses to care, as well as supporting clinical reasoning and communication between the members of the care team, ensuring continuity of care^(^ 25 ^)^.

In the Nursing Diagnosis as a Product and Interventions and Nursing domains, the scores in version I were close to the maximum level. It is noteworthy that, in both versions, the system offers support for clinical decision, suggesting diagnoses (with defining characteristics and related factors) and nursing interventions, and has mechanisms that prevent the user from progressing in the evaluation if the documentation is incomplete.

The new functionalities of version II with computerized scheduling of the nursing prescription contributed to the increase in the score in the Nursing Intervention domain, the possibility of recording the care provided alongside the prescription check. This contribution generated an unequivocal record of who performed the care and how they performed it. If any prescription item is not performed, the system warns the users that they need to justify why.

Compared to conventional paper documentation, electronic health records produce clear and legible data that lends itself well to coders, computational analyses and health service research studies^(^ 26 ^)^.

However, the development of effective systems to document the NP is certainly difficult in the area of health and nursing informatics. Structural problems such as lack of data standardization, safety mechanisms that prevent the user from progressing without completing every item, lack of adequate training and resistance to the adoption of these systems are pointed out as factors that affect quality and usability^(^ 26 ^)^.

In the Nursing Outcomes domain, the increase in the score can be attributed to the introduction of the Nursing Outcome Indicators (NOC) with measurement scales in version II of the system. The possibility of free text entry on the Summary screen, in the Nursing Evolution space, also contributed to the results achieved in this domain, providing a global view of the patient and the outcomes achieved.

This study supports the application of new concepts in Nursing, through the use of outcome indicators to determine a goal for the patient, the family or the community. Baseline results are measured at the initial assessment and progress is measured at each new assessment.

However, a difficulty in applying the NOC is the absence of operational definitions for Likert indicators and scales, which can lead to disparities in the interpretation of their scores. One of the proposed solutions to solve this issue is the development of studies through consensus among specialists to develop conceptual and operational definitions for the outcome indicators^(^ 27 ^)^.

In the same context, a study that clinically validated the nursing outcome indicators of Tissue Integrity: Skin and Mucous Membranes and their conceptual and operational definitions, concluded that the use of indicators with definitions can contribute to a reliable and accurate assessment of tissue integrity and assist in measuring the effectiveness of the nursing care provided^(^ 28 ^)^.

It is believed that the factors that contributed to the successful implementation of version II of the system are linked to the importance that the professionals attach to the NP in the institution, the efficacy of the system to document the NP and the effectiveness of the interventions related to user training and monitoring and system implementation with new features.

These results corroborate data from a systematic review, concluding that, although the initiatives implemented to improve the quality of documentation in the EHRs are really varied, the most successful interventions can be related to the training of the team and to the implementation of a new reporting system in EHR^(^ 29 ^)^.

The importance of educational interventions was also verified in a study in which the Q-DIO was used to measure the quality of nursing records at the pre- and post-intervention moments. This process concluded that they were important to improve the quality of nursing care documentation and visibility^(^ 30 ^-^ 31 ^)^.

As limitations of this study, it is pointed out that the data were collected immediately after the training and implementation of the second phase of the system. It is known that changes in the practice may require more time to develop.

It can be thought of the detection bias, since the researcher works with the system, and may in some way interfere in the evaluation of the outcome. There is also the previous literacy bias of the professionals, since they have used version I of the system since 2009. In addition, the institution has a strongly consolidated NP and continuously invests in training and development of the nursing team in clinical reasoning, critical thinking and in the use of the SLSs.

The results of this study can be used as reference data for the evaluation of computerized systems to document the NP, analysis of compliance of the documentation with the standards established in the literature and development of user-oriented systems. Such result contributes to the clinical practice, quality of care, visibility of Nursing as a profession, adequate dimensioning of nursing professionals, audit processes and assessments of care costs.

This assessment method can be applied in research studies to assess the quality of nursing records and audit processes, enabling adjustments to the NP documentation by means of educational interventions. These analyses will allow for periodic feedbacks to be offered to the nurses, in order to constantly support them in the practice of critical thinking and clinical reflection.

## Conclusion

The results of this research confirmed the study hypothesis. According to the total score of the Brazilian version of Q-DIO, the users documented the NP more effectively in PROCEnf-USP version II. There was a reduction in the percentage of items not documented in all the subscales evaluated. It was also possible to observe the effectiveness of the interventions, proving that team training and the deployment of a new system, with more functionalities, are factors that contribute to improve the quality of the NP documentation.
